# Monitoring the dead as an ecosystem indicator

**DOI:** 10.1002/ece3.7542

**Published:** 2021-05-01

**Authors:** Thomas M. Newsome, Brandon Barton, Julia C. Buck, Jennifer DeBruyn, Emma Spencer, William J. Ripple, Philip S. Barton

**Affiliations:** ^1^ School of Life and Environmental Sciences The University of Sydney Sydney NSW Australia; ^2^ Department of Biological Sciences Mississippi State University Mississippi State MS USA; ^3^ Biology and Marine Biology University of North Carolina Wilmington Wilmington NC USA; ^4^ Biosystems Engineering and Soil Science University of Tennessee Knoxville TN USA; ^5^ Department of Forest Ecosystems and Society Oregon State University Corvallis OR USA; ^6^ School of Science Federation University Australia Mt Helen VIC Australia

**Keywords:** carrion, decomposition, ecosystem health, indicators

## Abstract

Dead animal biomass (carrion) is present in all terrestrial ecosystems, and its consumption, decomposition, and dispersal can have measurable effects on vertebrates, invertebrates, microbes, parasites, plants, and soil. But despite the number of studies examining the influence of carrion on food webs, there has been no attempt to identify how general ecological processes around carrion might be used as an ecosystem indicator. We suggest that knowledge of scavenging and decomposition rates, scavenger diversity, abundance, and behavior around carrion, along with assessments of vegetation, soil, microbe, and parasite presence, can be used individually or in combination to understand food web dynamics. Monitoring carrion could also assist comparisons of ecosystem processes among terrestrial landscapes and biomes. Although there is outstanding research needed to fully integrate carrion ecology and monitoring into ecosystem management, we see great potential in using carrion as an ecosystem indicator of an intact and functional food web.

## Graphical abstract

We argue that information about the processes and organisms involved with the consumption and decomposition of dead animal matter (carrion) can provide insights into the broader health and integrity of ecosystems.

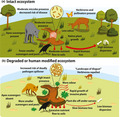

## 1 INTRODUCTION

Decomposition of dead plant and animal material has long been considered an integral component of ecosystems because it drives nutrient and energy flux (Lindeman, 1942). But the mechanisms by which dead animals, or carrion, affect ecosystems are only now being elucidated (Benbow et al., 2019). This presents new opportunities to investigate how carrion might serve as an integrative and focal resource for assessing key ecological processes and species interactions linked to ecosystem structure and function. Indeed, while current monitoring methods such as large‐scale camera trapping targeting vertebrates (e.g., Burton et al., 2015), or insects counts (e.g., Warren et al., 2021), can provide insights into the factors that influence animal abundance, they do not provide opportunities to assess how the presence or absence of different trophic guilds affects specific ecosystem processes. Monitoring vertebrate and insect scavengers at carcasses, on the other hand, allows for a direct assessment of how their presence or absence affects specific processes such as nutrient cycling or reduced biomass dispersion. Thus, there is a direct biodiversity to ecosystem process link.

**FIGURE 1:**
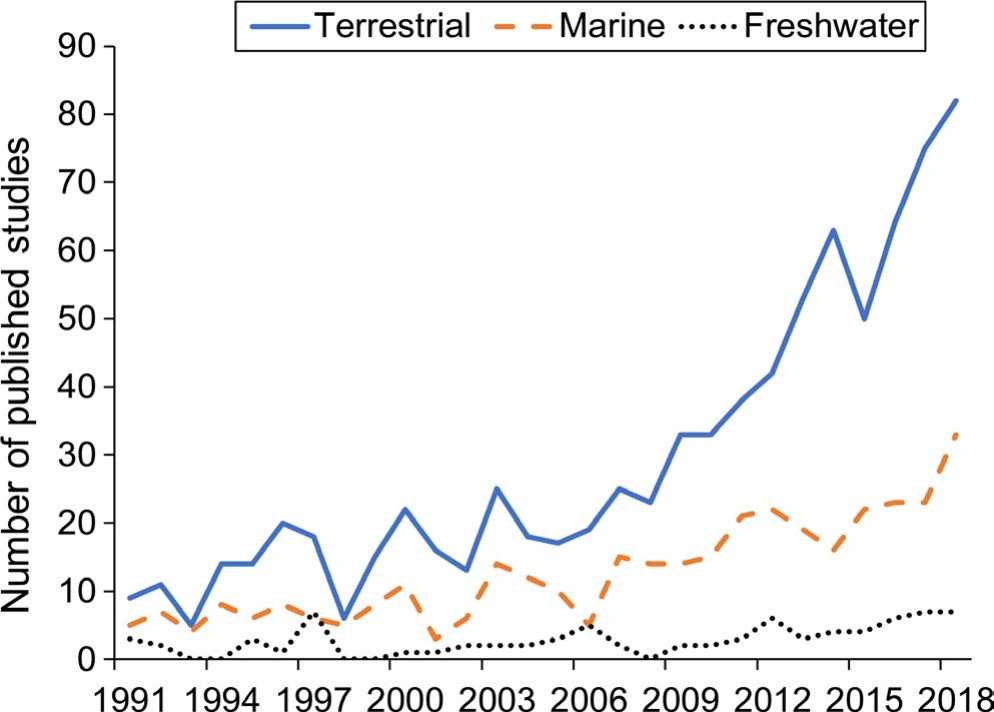
Trends in the number of articles published on carrion ecology. Articles in this figure are based on a Web of Science search for articles with topic (title, abstract, or key‐word) ‘carcass AND ecology’ OR ‘carrion AND ecology’ OR ‘scaveng* AND ecology’ between 1990 and 2018

Terrestrial ecosystems arguably offer the greatest potential to explore the use of carrion as an ecosystem indicator because this is where carrion research efforts have focused (Figure 1). The potential importance of carrion in terrestrial ecosystems is also reflected in the suggestion that more energy is transferred within terrestrial food webs as a result of scavenging compared to predation (Wilson & Wolkovich, 2011), and similarly that the role of carrion in nutrient cycling and community dynamics is disproportionately large compared with plant detritus (Barton et al., 2013). Indeed, carcasses are widespread and far more prevalent than often assumed, and linkages between individual carcasses, populations, communities, and ecosystems can be used to estimate carrion biomasses at each scale (Barton, Evans, et al., 2019). This reflects the fact that monitoring data from single carcasses can provide a small window into much wider processes happening at much larger spatial scales. Furthermore, carrion produced in terrestrial environments does not always stay there, and allochthonous carrion inputs can influence neighboring habitats. For example, the combination of hippopotamus (*Hippopotamus amphibious*) excretion/egestion and wildebeest (*Connochaetes taurinus*) carcass inputs have been shown to influence nutrient concentrations, nutrient limitation, and ecosystem function in a large African river (Subalusky et al., 2018).

The need to monitor carrion is further highlighted by the fact that human influence can alter ecosystem processes and pathways of energy transfer associated with carrion in terrestrial ecosystems (Mateo‐Tomás et al., 2019). For example, if larger scavengers are persecuted, and there is a subsequent decline of competition for carrion resources, carcasses could persist for longer and the remaining scavengers might face risk of pathogen exposure (O’Bryan et al., 2018). Alternatively, humans may subsidize scavenger communities via big game hunting and disposing of livestock carcasses (Mateo‐Tomás et al., 2015) or contribute to mass mortality events via eradication of unwanted animals (e.g., nuisance species or diseased animals) (Baruzzi et al., 2018). This can alter terrestrial ecosystem dynamics, especially if pest species like feral domestic dogs (*Canis familiaris*) increase in abundance as a result of increased carrion availability (Newsome et al., 2015).

**FIGURE 2:**
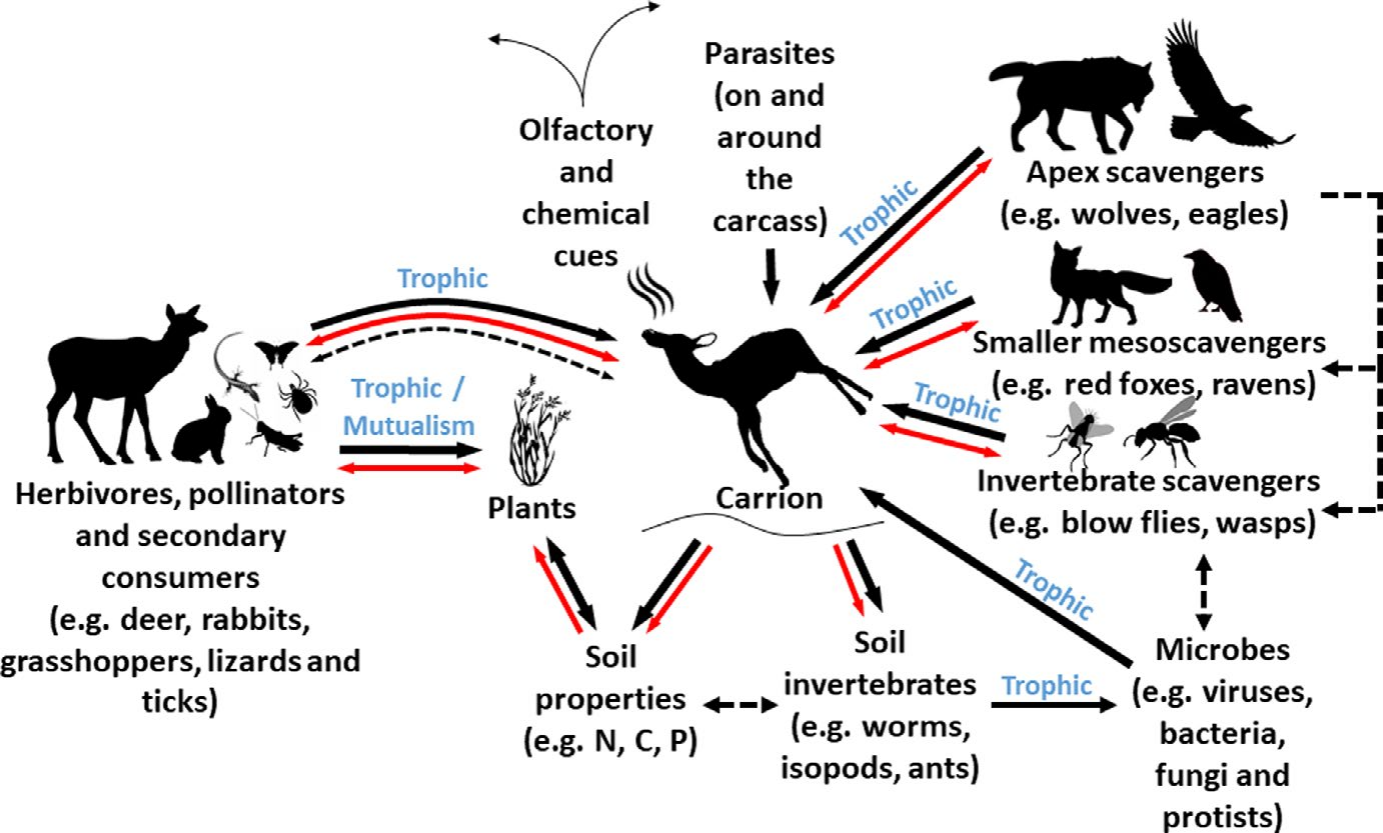
Web of interactions and ecological processes that take place around carrion in a terrestrial ecosystem. Carrion provides a food source and/or focal point of attraction for vertebrate and invertebrate scavengers. Carrion decomposition influences soil biogeochemistry and below ground invertebrates. The vegetation that grows following decomposition influences use of the area by herbivores and pollinators. Large scavengers are likely to influence carrion decomposition rates and use of carrion by smaller scavengers because they can rapidly consume carrion and exclude other scavengers through direct predation and/or fear effects. Parasites and microbes may deter consumption by scavengers, kill scavengers in the case of deadly pathogens, and/or deter herbivores from foraging near carcasses. Secondary consumers are attracted to carrion because of the presence of scavengers that are prey. Black solid arrows indicate use of carrion, vegetation, microbes, or scavengers (as prey). Black dotted arrows indicate the potential for positive or negative interactions between different trophic groups. Red solid arrows indicate the possible microbial or parasite transmission/interaction pathways. Trophic interactions include feeding interactions. Mutualism includes interactions that benefit two or more species. Olfactory and chemical cues are volatile organic compounds that are released into the air from carrion. All of these biota, interactions, and processes are measurable. The absence of major changes to any of these key players and processes in carrion decomposition might be indicative of ecosystem dysfunction

We suggest that multiple interactions and ecological processes can be studied around carrion in terrestrial ecosystems and that carrion could be used as a tool to assess ecosystem structure and function (Figure 2). We herein develop a framework to capture this idea and discuss the strengths and weaknesses of our approach. We start by identifying potential indicators that could be monitored around carrion. We separate our indicators into two main groups: (a) scavenger indicators and (b) ecological indicators. Scavenger indicators, broadly defined, are linked to the consumption of carcasses by different scavenger guilds and how far they may disperse carrion nutrients via scat deposition. Ecological indicators reflect the consequences of carrion persistence or decomposition rates. We then provide suggestions on (a) how new research could progress the use and validation of our indicators and (b) preliminary options for resource managers and researchers to assess whether an ecosystem has an intact and functional food web that supports important elements of biodiversity using a subset of indicators. Our framework is intended to start conversations about how carrion monitoring can be better integrated into ecosystem management and whether carrion could be used as a tool to compare ecosystem structure and function around the world.

## 2 SCAVENGER INDICATORS

The role of carrion in shaping terrestrial food web dynamics varies depending on its quantity and dispersion within an ecosystem, habitat context, season, climate, anthropogenic impacts, scavenger community composition, scavenger densities, and predation risk (Table S1). Nevertheless, a functioning ecosystem typically includes organisms that recycle carrion efficiently and quickly. Conversely, animal carrion that is not recycled quickly or efficiently might be evidence of a degraded ecosystem. The presence or absence of different scavengers is therefore important to consider, with each scavenger group fulfilling key roles linked to ecosystem structure and/or function (Table 1).

**TABLE 1:** Scavenging observations that can be used as indicators of ecosystem structure and function

Observation	Main Link to Ecosystem Structure and Function	Potential method of measurement and associated metrics	Example	Strength/Weakness
Apex and mesoscavengers	Whether an ecosystem has an intact and functional food web, whether there is likely to be efficient removal of carrion tissue and bones which accelerates biogeochemical cycling and regulates carrion availability to other scavengers.	Cameras on carrion to measure relative use of carcasses by apex and mesoscavengers, carrion, and bone persistence rates, and interactions and contact rates to assess competition and fear effects.	Cunningham et al. (2019)	*Strength:* Cameras are commonly used and a cost‐effective way to study vertebrate use of carcasses. *Weakness:* Large amounts of data to process.
Invertebrate scavengers	Whether an ecosystem has an intact and functional food web that includes invertebrate scavengers that can accelerate decomposition.	Cameras, pitfall, and sticky traps, sweep nets, manual collection from carrion to measure invertebrate presence, abundance, diversity, and richness as well as interactions with other species.	Farwig et al. (2014)	*Strength:* Invertebrates can be easily and cost effectively sampled around carcasses. *Weakness:* Taxonomic expertize is needed.
Microbes	Whether an ecosystem has a diverse microbial community; diverse communities are more likely to have a variety of saprotrophs and maintain functions during the decomposition disturbance. The presence of pathogenic microbes indicates disease spillover risk.	Molecular microbial ecology approaches, for example, swabbing carcasses to sample microbes at set intervals and identifying them using 16S rRNA amplicon sequencing or other omic approaches; qPCR quantification of populations or functional genes of interest.	Maron et al. (2018)	*Strength:* Methods are established and could be used to identify pathogens. *Weakness:* Costs and expertize can be prohibitive.
Invasive scavengers	Whether an ecosystem is supporting invasive scavengers or invasive pathogenic microbes, and how invasive scavengers can influence energy flow through food webs.	Cameras, pitfall, and sticky traps, sweep nets, manual collection from carrion to measure invertebrate presence, abundance, diversity, and richness as well as interactions with other species. Molecular microbial ecology approaches (as above) for pathogens.	Abernethy et al. (2016)	*Strength:* Sampling methods for apex, meso, invertebrate scavengers, and microbes can be used to detect invasive species. *Weakness:* Costs and expertize can be prohibitive.

### 2.1 Apex scavengers

**FIGURE 3:**
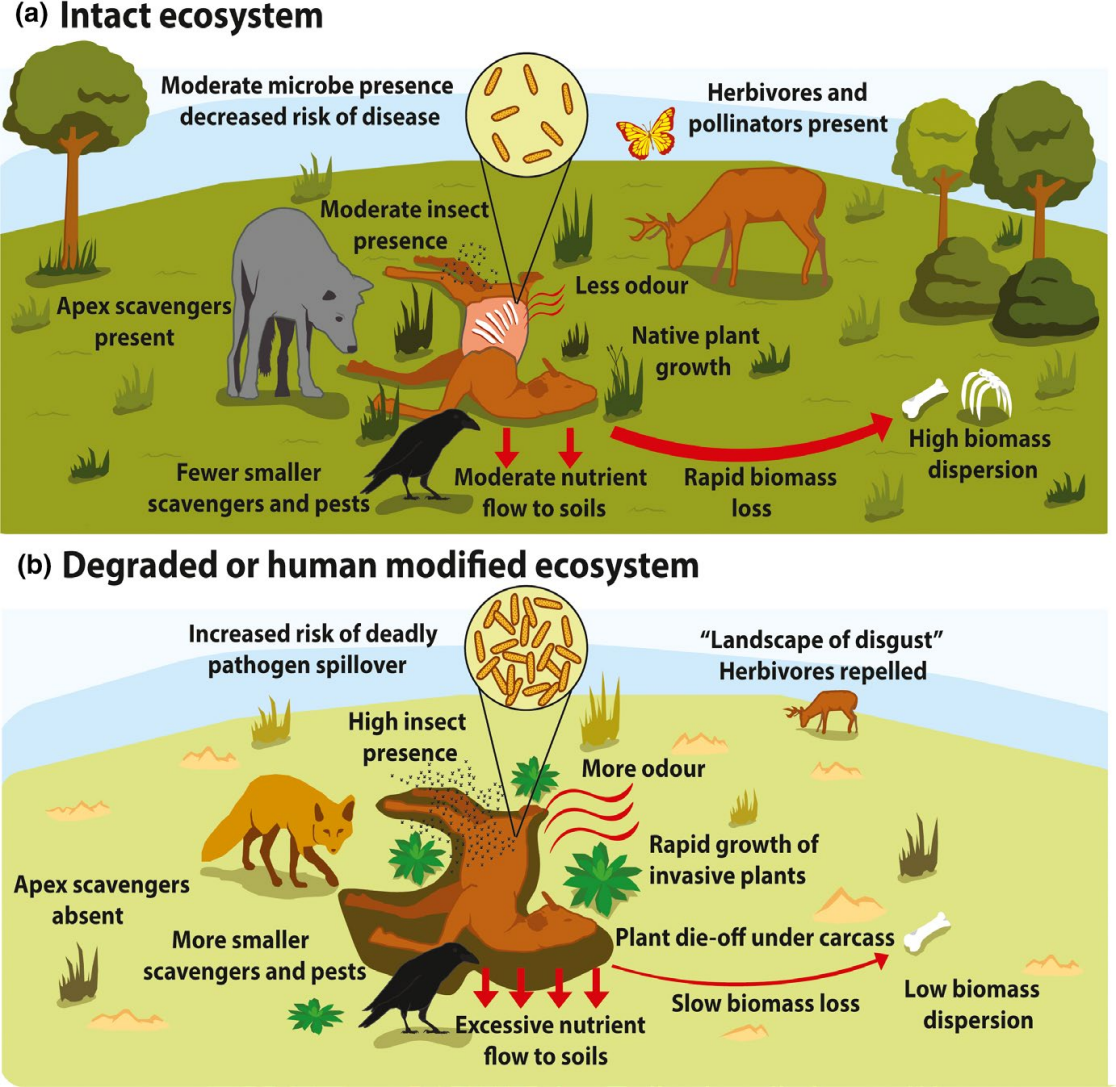
Hypothetical depiction of what could potentially happen around carrion in an (a) intact ecosystem and (b) degraded or human‐modified ecosystem. Each of the responses we note (text in figures) is relevant to our proposed scavenger and ecological indicators. The figures reflect that apex scavengers are present in an intact ecosystem, whereas mesoscavengers, insects, and introduced species dominate use of carrion in the degraded ecosystem. As a result, carrion biomass loss and dispersion should be greater in the intact ecosystem. The proportional change in soil properties (e.g., total nitrogen and carbon content) during decomposition should be lower in the intact ecosystem. Key soil invertebrates may be absent from degraded ecosystems and thus be absent under carrion. Plant die‐off and invasive plants are likely to be more prevalent under and around a carcass following excessive carcass nutrient flow to soils in the degraded ecosystem. Plant die‐off and the lack of native vegetation could impact native herbivores. However, the greatest impacts to herbivore species will likely come as a result of increased odor resulting from a lack of any functional scavenger guild in degraded ecosystems. Deadly pathogens may become prevalent in degraded ecosystems, leading to increased spillover risks around carrion. This may create a “landscape of disgust” and will also repel herbivores from the local area (see Weinstein et al., 2018)

Apex scavengers are dominant scavengers as determined by their ability to locate and rapidly consume carrion including bones. Obligate scavengers like vultures, which rely solely on carrion to meet their energetic demands, have disproportionately large effects on carrion persistence compared to smaller scavengers and microbial decomposers (Ogada et al., 2012). However, large facultative scavengers (predators that take advantage of opportunities to feed on carrion, such as wolves) can also rapidly consume carrion and bones and can therefore be considered apex scavengers. Because apex scavengers disperse nutrients via their scats, their absence could increase nutrient loads around carrion (Barton, Cunningham, Lindenmayer, et al., 2013; Benbow et al., 2019). The presence of apex scavengers at a carcass also determines whether other species utilize it because smaller scavengers must optimize the risk of injury or death to a larger competitor prior to attempting consumption (so‐called “fear” effects) (Atwood & Gese, 2010). The loss of apex scavengers could therefore alter food web dynamics around carrion, while their presence indicates an intact and functional scavenger guild (Figure 3).

### 2.2 Mesoscavengers

Mesoscavengers, such as red foxes (*Vulpes vulpes*), racoons (*Procyon lotor*), and ravens (*Corvus corax*), typically consume less carrion than apex scavengers because they are smaller in size and have lower energetic needs (Mateo‐Tomás et al., 2017). This means carrion persists longer when mesoscavengers dominate its use (Cunningham et al., 2019). Increases in mesoscavenger use of carrion can trigger indirect effects in the surrounding landscape, including elevated predation by facultative mesoscavengers on ground‐nesting birds (Cortés‐Avizanda et al., 2009), increased disease risk (Markandya et al., 2008), and changes to community structure (Sebastián‐González et al., 2016). The dominance of mesoscavengers at carrion sites could also indicate that an ecosystem has undergone a regime shift to favor smaller scavengers. This could occur under scenarios where there is reduced competition for carrion resources, such as following apex scavenger declines (O’Bryan et al., 2019), or when there are large carrion loads in the landscape (Newsome et al., 2015). If these conditions result in elevated numbers of mesoscavengers, there could be unintended and indirect effects on other species and ecological processes, akin to those reported following increases in mesopredator populations (Prugh et al., 2009). Conversely, a reduction in mesoscavengers could also be indicative of a degraded ecosystem; experimental elimination of mesoscavengers has been shown to reduce scavenging efficiency compared to an intact scavenger community and increase resources going to other scavengers (Turner et al., 2020).

### 2.3 Invertebrate scavengers

Invertebrates form the most diverse component of the nonmicrobial community at carrion sites (Benbow et al., 2019). In most terrestrial ecosystems, invertebrate scavengers rapidly consume dead animal matter (Barton & Evans, 2017). The important functional role of invertebrates has been demonstrated by the substantial delay in biomass loss when they are prevented from accessing carcasses (Payne, 1965). Blowflies (*F*. *Calliphoridae*), in particular, are efficient at removing soft tissues even from larger carcasses. Where carrion persists for long periods due to vertebrate scavenger population declines or large carrion inputs, invertebrate scavenger abundance and/or diversity on carrion may increase. In this case, invertebrate scavengers could play a greater role in the breakdown and dispersal of carrion biomass (Barton, Cunningham, Lindenmayer, et al., 2013), and in some cases, they may outcompete vertebrate scavengers. However, invertebrates can never fully replace the functional roles of vertebrate scavengers because they are unable to rapidly consume bones or disperse nutrients to the same extent. Knowledge of the presence, abundance, and diversity of key invertebrate taxa at carrion sites is therefore useful to indicate whether an ecosystem has an intact and functional scavenger community (Figure 3).

### 2.4 Microbes

Decomposition is microbially mediated, with both the host and environmental microbial community playing key roles (Benbow et al., 2019). Shortly after death, microbes concentrated in the gastrointestinal tract and lungs begin to convert the products of autolyzing host cells (Javan et al., 2016; Lauber et al., 2014). Much of these early internal processes are anaerobic, producing gasses and nutrient‐rich liquids that ultimately are released to the ecosystem. Environmental microbes also colonize the carcass to aid in the breakdown (Lauber et al., 2014). In terrestrial ecosystems, released decomposition products cause pronounced changes to soils: fluids introduce a mix of nutrients and labile organic compounds, stimulating microbial activity (Keenan et al., 2018). In addition, decomposition fluids saturate soils creating anaerobic conditions and altered pH, which affects both the types and activities of microbes (Metcalf et al., 2016) and microfauna (Taylor et al., 2020) and thus controls nutrient cycling (Keenan, Schaeffer, et al., 2018). Diverse microbial communities are more likely to have a variety of heterotrophic saprotrophs (i.e., microbes that process decayed organic matter) and maintain community functions during decomposition. The composition of microbial communities may be informative as an indicator, with soil fungal versus bacterial dominance, or the presence of particular functional groups, indicative of decomposition stage. For example, studies of soil microbial community succession during carrion decomposition have consistently reported increased prevalence in obligate and facultative anaerobes such as Firmicutes (Cobaugh et al., 2015; Metcalf et al., 2016; Pechal et al., 2013).

In addition to the saprotrophic microbes, it is possible for pathogenic or toxin‐producing organisms to proliferate on carcasses. For example, *Clostridium* spp. can proliferate in decomposing tissues (Hyde et al., 2013) as well as in the soils affected by decomposition fluids (Cobaugh et al., 2015). It is thought that pathogens with environmental reservoirs may employ a “microbial mutiny” strategy in which they begin proliferating in an aging or sick host to get a head start on decomposition and ensure their survival and transmission in the decomposition environment (Rózsa et al., 2017). Toxin‐producing organisms may also be present; for example, *Clostridium botulinum* proliferates in carcasses after death and produces botulinum toxin, which is lethal to vertebrates that ingest it (Espelund & Klaveness, 2014). The presence of pathogenic microbes can present health risks to livestock and humans.

### 2.5 Invasive species

Carrion could support populations of invasive vertebrate and invertebrate species and exacerbate their impacts on ecosystems under some circumstances. Their presence might also influence nutrient flow through food webs. For example, invasive fire ants (*Solenopsis invicta*) can deter scavengers from consuming carcasses by predating on carrion‐feeding invertebrates and by altering the microhabitat of carrion via nest and mound construction, thus restructuring and slowing the process of decomposition (Eubanks et al., 2019). Alternatively, invasive species may rapidly find and remove carrion in some environments; on three islands in Hawaii, invasive scavengers removed 55% of monitored carrion (Abernethy et al., 2016). Carrion or scat deposited by scavengers could also spread pathogenic microbes such as African swine fever into new regions (Probst et al., 2017). This could impact native animal populations, humans, and livestock. In addition, invasive herbivores could produce large pulses of carrion, as they can achieve high abundances, and their populations can experience large die‐off events due to starvation, disease, droughts, and floods or through direct killing by humans. Such events could contribute substantially to the energy budgets of invasive scavengers, and it is unknown whether native scavengers can rapidly consume unusually high carrion inputs stemming from invasive populations. Although invasive species may utilize carrion and thus help to recycle its nutrients, dominant use of carrion by invasive species still indicates that the area has a stable population of invasive species and that carrion is supporting them, potentially exacerbating their impacts and altering the way energy flows through scavenging food webs.

## 3 ECOLOGICAL INDICATORS

There are several ecological processes that stem from the release of nutrients from carrion into surrounding soils. Animal behavior might also be influenced by the presence of infectious agents or olfactory risk cues around carrion. Such effects are ultimately linked to how long carrion persists in the environment and can have both positive and negative impacts on ecosystems. The ecological processes that occur around carcasses are impacted by biophysical factors (Table S1), and these must be taken into account. Nonetheless, the following ecological indicators could be useful to monitor in addition to the presence or absence of different scavengers around carrion (Table 2).

**TABLE 2:** Ecological observations in or around carrion that can be used as indicators of ecosystem structure and function

Observation	Link to Ecosystem Structure and Function	Method of measurement and associated metrics	Example	Strength/Weakness
Carrion biomass and dispersion	Contribution to trophic processes and ecosystem stocks and flows as well as understanding the relative contribution of scavenging versus predation in ecosystems and how energy flows through food webs.	Time to carrion removal or proportion, weight, or type of bones remaining following decomposition. Quantities derived from individual carcasses (body mass, consumers, and rates of decay) can be scaled up using population metrics to infer carrion biomass at the ecosystem level. Scavenger movement and diet responses to changes in carrion biomass can be accessed via tracking and scat analyses to assess dispersion.	Barton, Evans, et al. (2019)	*Strength:* Biomass loss can be inferred easily via observation, cameras, or by weighing the carcass at set intervals. *Weakness:* Dispersion studies would be intensive and expensive.
Soil biogeochemistry	Functional ability of scavengers to remove carrion which influences nutrient transfer from carrion to soils.	Carbon, nitrogen, phosphorus, and pH levels in soils before, during, and after decomposition.	Keenan, Schaeffer, et al. (2018)	*Strength:* Soil analyses are well established and can be easily collected under carcasses. *Weakness:* Costs and expertize can be prohibitive.
Soil invertebrates	Trophic processing of carrion tissues and elevated bacterial loads that enter the soil profile.	Densities of soil nematodes and other invertebrates at carcass decomposition hotspots.	Szelecz et al. (2016)	*Strength:* Samples can be easily collected under carcasses. *Weakness:* Taxonomic expertize is needed.
Vegetation responses	Functional ability of scavengers to remove carrion which influences nutrient transfer from carrion to soils and subsequent vegetation responses.	Abundance, richness, and diversity of vegetation before, during, and after decomposition.	Barton et al. (2016)	*Strength:* Vegetation can be easily sampled around carcasses at set time intervals. *Weakness:* Taxonomic expertize is needed.
Herbivores, pollinators, and secondary consumers	Modifies willingness of animals to forage near carcasses which influences grazing and pollination regimes. Presence of secondary consumers indicates broader role of carrion in ecosystem.	Herbivore movement and behavioral responses to carrion presence.	Baruzzi et al. (2018) and Weinstein et al. (2018)	*Strength:* Behavioral responses in close proximity to carcasses can be inferred easily from cameras. *Weakness:* Movement and behavioral responses at the landscape scale would be labor‐intensive and costly.
Infectious agents	Modifies willingness of animals to scavenge, thereby influencing carcass persistence.	Abundance, diversity, and richness of endo‐ and ectoparasites and microbial communities (viral, bacterial, fungal, and protist), during, and after decomposition. Evidence of deadly pathogen spillover to humans and wildlife.	Benbow et al. (2019)	*Strength:* Methods are established and could be used to identify infectious agents. *Weakness:* Costs and expertize can be prohibitive.
Olfaction	Altered VOC profiles or absent VOCs may influence rates of scavenging and in turn carrion persistence.	Measurements of VOC profiles around carrion during the different stages of decomposition.	Grigg et al. (2017)	*Strength:* Methods are established from forensic studies and could be applied in the field. *Weakness:* Specialist equipment required.

### 3.1 Carrion biomass loss

The dispersal of nutrients from carrion occurs across different scales. Locally, carcass fluids deliver nutrients into the soil (Keenan, Schaeffer, et al., 2018). The consumption of soft tissues by insects and their development and pupation also recycles nutrients locally. The dispersal of insects and defecation by vertebrate scavengers away from carcasses delivers nutrients further into the landscape (Bump et al., 2009; Hocking & Reimchen, 2006). Temporally, soft tissues are consumed most rapidly, and their nutrients are also recycled quickly (hours/days) (Barton et al., 2019). Tissues such as bone, hair, and nails/hooves break down more slowly (days/weeks/months). For example, skeletal remains can deliver phosphorus and calcium into the environment many weeks to months after soft tissues have been removed (Melis et al., 2007). Relevant indicators to consider include the *rate* of biomass loss (i.e., consumption rates) and the *completeness* of carcass removal (soft tissues only or entire carcass). These are related to scavenger use of carcasses and the time it takes for scavengers to locate carcasses. A slow rate of biomass loss might indicate that an ecosystem has lost a functional scavenger guild (e.g., Turner et al., 2020) or that there are excessive carrion biomass loads and scavengers are overwhelmed. Conversely, complete and rapid carcass removal and recycling indicates the presence of key vertebrate scavengers that can consume both tissue and bones (Figure 3).

### 3.2 Soil biogeochemistry

In the early stages of decomposition, soil moisture, nitrogen/ammonium, pH, and electrical conductivity can change under a carcass (Keenan, Schaeffer, et al., 2018; Quaggiotto et al., 2019). Later stages of advanced and dry decomposition can affect phosphorus and nitrate levels in soils. The most obvious changes can be summarized as a nutrient or fertility “hotspot” being created by elevated nitrogen and other macronutrients (Quaggiotto et al., 2019). Indicators of soil changes include decreased C:N ratio. Temporal indicators of persistence might include phosphorus concentrations or N isotopic ratios (Keenan et al., 2019) as these take longer to return to background levels. Studies at human decomposition facilities that practice repeated concentrated deposition of cadavers have shown that increased carrion loads significantly alter soil biogeochemistry (Damann et al., 2012). Major changes to soil properties will potentially reflect excessive nutrient loads flowing into surrounding soils which could, in turn, impact associated biological community structure and function.

### 3.3 Soil invertebrates

Nematodes and soil mites accumulate under carcasses to exploit microorganisms. The proliferation of bacterial‐ and fungal‐feeding nematode taxa, in particular Rhabditidae, has been observed (Keenan, Emmons, et al., 2018; Szelecz et al., 2018; Taylor et al., 2020). Soil nematodes are a major part of belowground food webs, and their accumulation under carcasses could indicate soil bacterial loads, with further important ramifications for soil food webs. Other soil microfauna that are part of the decomposition food web include mites (Szelecz et al., 2018), amoebae (Seppey et al., 2016), collembola (Klonowski et al., 2015), and tardigrades (*Hypsibius* spp.) (Keenan, Emmons, et al., 2018).

### 3.4 Vegetation responses

**FIGURE 4:**
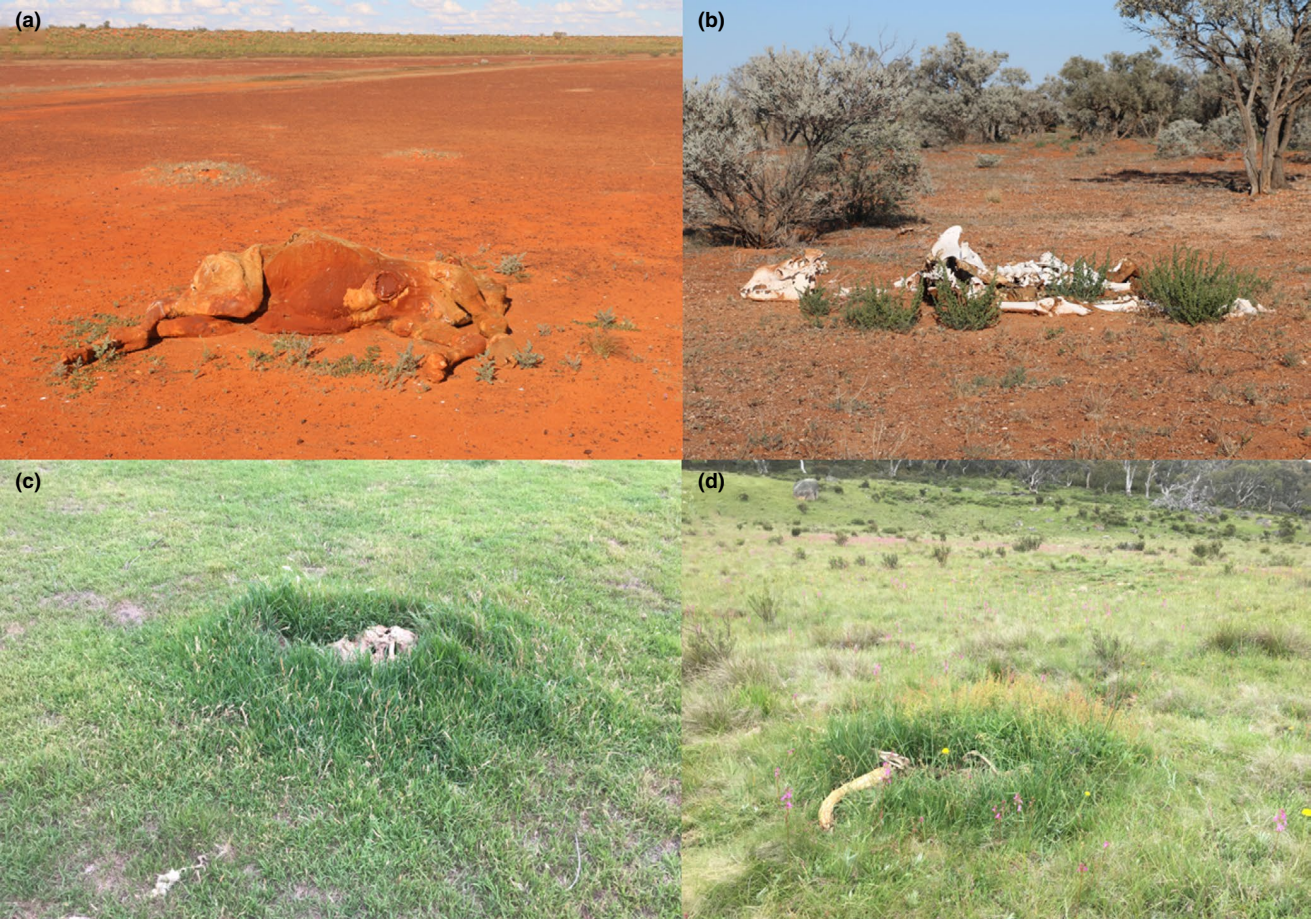
Plant growth responses to the presence of camel carcasses in the Simpson Desert, central Australia (a and b), and the presence of kangaroo carcasses in the Blue Mountains (c), and Kosciuszko National Park (d) eastern Australia

Plants respond to elevated nutrient concentrations through enhanced growth and floristic responses (Figure 4), and therefore represent a potential indicator of the consequences of nutrient flow into soil. Nutrient tolerant, “weedy,” or opportunist species may gain a competitive advantage over native or nutrient‐sensitive species following decomposition (Barton, Cunningham, Macdonald, et al., 2013; Barton et al., 2016), and they may be indicative of prolonged decomposition and excess nutrient loads in soils, or potentially even changes to microbial communities within soils.

### 3.5 Herbivores, pollinators, and secondary consumers

Vegetation that grows around carrion sites can have higher nitrogen content following decomposition (Barton, Cunningham, Macdonald, et al., 2013) and may attract herbivores, illustrating the potential for cascading trophic effects of carrion on food web dynamics (Figure 2). Carrion can also support pollination services provided by flies (Cusser et al., 2020). Measurements of carrion dispersion and biomass (Barton, Evans, et al., 2019) could allow useful predictions about the overall effects carrion has in shaping the behavioral and foraging dynamics of herbivores and pollinators. In addition, carrion can attract secondary consumers like insectivorous vertebrates (e.g., lizards, passerine birds, armadillos) and invertebrates (e.g., hornets, parasitoids), as well as micropredators (e.g., mosquitoes) and parasites (e.g., ticks, bot flies). Knowledge of secondary consumer presence, abundance, and diversity could similarly provide insights into the broader role of carrion in ecosystems.

### 3.6 Infectious agents

Vertebrate carcasses may harbor a variety of pathogens and parasites at the time of death, and others that colonize and proliferate after death. Some of these infectious agents can be transmitted to scavengers via trophic transmission or direct contact. For example, *Bacillus anthracis, Clostridium perfringens, Escherichia coli, Staphylococcus aureus, Shigella dysenteriae, Salmonella typhi, Francisella tularensis,* rabies, tuberculosis, chlamydia, cysticercosis, *Echinococcus*, prion diseases, and a variety of ectoparasites could be transmitted via scavenging. Therefore, infectious agents represent integral components of the carrion food web (Figure 2) and their presence could indicate ecosystem integrity (Hudson et al., 2006) and a functional scavenger guild (Figure 3).

Alternatively, if one or a few pathogen‐resistant scavengers consume a carcass quickly, this can reduce opportunities for transmission to other species (Ogada et al., 2012), thereby suppressing disease. For instance, vultures possess physiological adaptations such as extremely acidic stomachs that readily kill most bacteria and viruses, and they are generally more resistant than other scavengers to the toxins produced by decomposers (Roggenbuck et al., 2014). Indeed, the decline of vulture populations and high human population density in South Asia has been linked with increases in incidences of bubonic plague and rabies (Ogada et al., 2012; Wandeler et al., 1993). Therefore, disease outbreaks could indicate that an ecosystem is lacking a functional scavenger guild (Figure 3).

### 3.7 Olfaction

Animals communicate with their environment through the use of chemical mediators and olfactory risk cues (Haswell et al., 2018). Scavengers, including necrophagous insects and new world vultures, are attracted to volatile organic compound (VOC) cues which are released by carrion at different stages of decomposition (Dekeirsschieter et al., 2009). Altered, concealed, or absent VOC profiles at carcasses may not attract scavengers rapidly and could prolong carrion persistence, with consequences for rates of nutrient flow or pathogen risk.

## 4 OPTIONS FOR MONITORING THE DEAD

We have suggested a suite of indicators that can provide information about carrion and its role in terrestrial ecosystems, as well as the ability of ecosystems to function efficiently and recycle heterotrophic biomass. The diverse set of indicators detailed here could be used to infer ecosystem structure and/or function based on rates of carrion removal, the presence or absence of key scavenger species (Table 1), and ecological interactions and processes (Table 2). Scavenger indicators might be examined at carcasses at landscape scales (multiple carcasses), whereas trophic linkages, nutrient cycling, and insect biodiversity can be monitored at local scales (individual carcasses). The range of indicators to monitor provides many options to examine decomposition processes in ways that suit different biomes and land management needs. However, to put this plan into action, we suggest two research priorities.

The *first* research priority is developing baseline measures of scavenging and decomposition rates to facilitate comparisons within and among ecosystems. Determining the exact threshold values for what constitutes a healthy or degraded ecosystem may not be achievable in the first instance due to interacting factors we have identified that may contribute to variation in results (Table S1). However, the initial baseline measures can be used for comparison with future data to identify trends or changes. Research needs to be undertaken, therefore, to generate data that sets baseline expectations for carrion removal and decomposition rates in a variety of natural and modified systems. Scavenging rates can be assessed via a number of approaches including time to removal which is typical for smaller sized carcasses (e.g., Huijbers et al., 2015; Inger et al., 2016), or via monitoring carcasses using remote cameras that allows both monitoring of the scavengers themselves and the carcass (e.g., Cunningham et al., 2019). The exact contribution of different scavenger guilds to carcass removal can be assessed by simultaneously monitoring carcasses that are exposed to different scavenger exclusion scenarios (e.g., Bellan et al., 2013; Hill et al., 2018; Turner et al., 2020). Exact decomposition rates can be assessed by mass loss rates, measured visually or by weighing carcasses over time or at the end of an experiment. This method would work fairly easily for small carrion, but becomes more difficult with larger carcasses. However, decomposition can also be assessed using a visual scoring of morphological changes associated with decomposition, such as the total body score (TBS) method, originally developed for assessing human decomposition progression for forensic purposes (Megyesi et al., 2005) and adapted for domestic pigs (*Sus scrofa*) used as human analogs in decomposition research (Keough et al., 2017). The advantage of this method is that it can be done remotely using a time series of photographs of the decomposing carcass (Ribéreau‐Gayon et al., 2018). The challenge of applying the TBS method to carrion is that scoring can be dependent on the observer and would need to be validated for use on different animal species.

**FIGURE 5:**
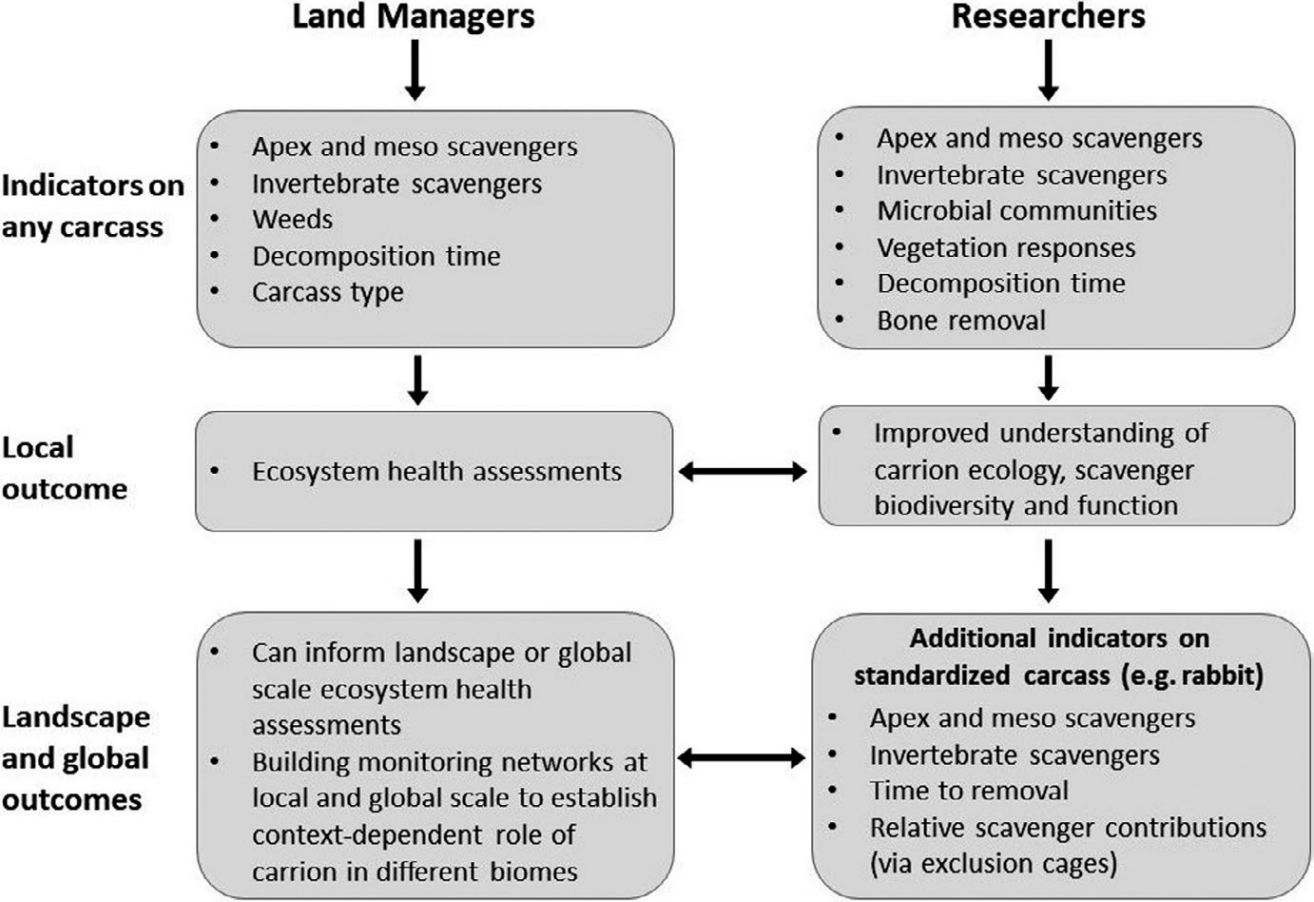
Overview of key ecosystem indicators that could be used by land managers and researchers when monitoring or studying carrion. The set of indicators will help inform ecosystem management at the local, landscape, or global level and could help form the basis of developing more detailed carrion monitoring networks. Additional indicators measured by researchers at a standardized carcass type could build knowledge of the context‐dependent role of carrion in a global range of biomes and provide the basis for scaling the work up to using larger carcasses and additional indicators

The *second r*esearch priority is to determine the most relevant indicators to prioritize. This will require consideration of the monitoring objectives, costs, technical expertize available, and any health risks associated with handling carcasses (Tables 1 and 2). For land managers, one feasible option might be to use fresh carrion (as a result of natural deaths, carnivore kills, or animals shot) when found in the landscape. In addition, a core set of indicators could be monitored in a cost‐effective manner without the need to handle the carcass or be exposed to any potential pathogens, including (a) presence/diversity of apex scavengers or mesoscavengers using remote cameras, (b) presence/diversity of invertebrate scavengers using pitfall or sticky traps, (c) presence of weedy plants following decomposition, and (d) the time to complete decomposition (Figure 5). Degraded ecosystems where there are active conservation management plans in place (e.g., controlling introduced species, protecting apex scavengers, or weed control) would be ideally suited for this type of monitoring because the results should help inform the effectiveness of the management interventions. In the first instance, we suggest that only a small number of carcasses (5–10) might need to be monitored by an individual to provide basic answers about what happens to carcasses in a given landscape. Over time, this approach lends itself toward community‐level involvement, whereby land managers across the broader landscape monitor many carcasses and make data publicly available to inform government‐led management initiatives (Figure 5).

For researchers interested in monitoring carrion, we suggest that a minimum set of indicators be measured in future studies including (a) presence/diversity of apex/meso vertebrate scavengers, (b) presence/diversity of invertebrate scavengers, (c) some measure of microbial diversity (sampled with appropriate personal protective gear and storage/transport options where pathogens are of concern), (d) basic soil chemistry (e.g., carbon, nitrogen, phosphorous), (e) vegetation responses, (f) the time to complete decomposition and (g) bone removal (Figure 5). If adopted, carrion would start to become a focal and/or consistent substrate for examining key ecological processes, akin to other global networks that focus on plant‐derived substrates, such as the tea‐bag index (Keuskamp et al., 2013) and other global nutrient networks (Stokstad, 2011).

We acknowledge there are challenges associated with using carrion as a standardized substrate in a global monitoring network. However, there are at least 44 sites globally that have recorded vertebrate scavengers on carrion, and efforts are underway to describe the factors influencing global vertebrate scavenging dynamics (Sebastián‐González et al., 2019). The next obvious step is to expand these efforts to include simultaneous measurements of a broader set of indicators, as listed above. In terms of a fully standardized approach, one option would be to focus on smaller carrion like rabbits or hares that are experimentally placed out across representative biomes. Time to removal would provide an indication of scavenger efficiency, the dominant scavengers could be recorded via remote cameras, and metal cages could be placed over a subset of rabbits to exclude vertebrate scavengers and assess the relative contribution of insects to decomposition. Once there is enough baseline data across a variety of land uses and habitats, options for scaling the work up to larger carcasses, or including additional indicators linked to soil, microbes, and vegetation communities could be incorporated (Figure 5). Depending on the size of the larger carcasses, they could be sourced and experimentally placed out, or monitored when found in the landscape (as a result of natural deaths, carnivore, or hunter kills). In terms of sample sizes (number of carcasses) needed to generate useful insights at a global scale, the data incorporated into Sebastián‐González et al. (2019) included studies with sample sizes ranging from 6 to 267, with an average of 49. This suggests that global insights can be gained from carrion studies with variable sample sizes, but also that studies with relatively small sample sizes can still be useful.

## 5 CONCLUSION

We see great potential to incorporate carrion monitoring into ecosystem management. To this end, we have reviewed a broad set of indicators of carrion consumption, decomposition, dispersal, and its consequences for ecosystems. These indicators provide a potential way to gain insight into scavenger diversity, abundance, and behavior around carrion, as well as assessments of the way energy flows from carrion into the surrounding environment. Together, these indicators could help to show if an ecosystem has an intact and functional food web. Monitoring carrion as an indicator of ecosystem structure and function has both strengths and weaknesses, but to start the conversation, we have outlined options for land managers and researchers to pursue (Figure 5). If taken up, it would allow for the development of baseline datasets on scavenger species, carrion decomposition rates, behaviors and interactions, soil and plant responses, and disease presence and risk. It would also set the stage for carrion to be used more widely as an indicator to assess how changes to ecosystems, including biodiversity loss, will impact ecosystem structure and function.

## CONFLICT OF INTEREST

None declared.

## AUTHOR CONTRIBUTIONS

**Thomas Newsome:** Conceptualization (equal); writing–original draft (equal); writing–review and editing (lead). **Brandon Barton:** Conceptualization (supporting); writing–original draft (supporting); writing–review and editing (supporting). **Julia Buck:** Conceptualization (supporting); writing–original draft (supporting); writing–review and editing (supporting). **Jennifer DeBruyn:** Conceptualization (supporting); writing–original draft (supporting); writing–review and editing (supporting). **Emma Spencer:** Conceptualization (equal); writing–original draft (supporting); writing–review and editing (supporting). **William J. Ripple:** Conceptualization (supporting); writing–original draft (supporting); writing–review and editing (supporting). **Philip Barton:** Conceptualization (equal); writing–original draft (equal); writing–review and editing (supporting).

## Supporting information

Supplementary Material

## Data Availability

Data for Figure 1 come from publicly available sources without restriction. No other data are included in this paper.
