# Supplementary material for: Monitoring the dead as an ecosystem indicator
Source: Ecol Evol. 2021 May 1;11(11):5844–56. doi: 10.1002/ece3.7542 (PMC8207411; doi:10.1002/ece3.7542)
Supplement: Supplementary file 1 — Supplementary Material [file ECE3-11-5844-s001.docx]

**Supplementary Material**

**Table S1:** A selection of key biophysical factors that may impact carrion indicator metrics. Potential methods of measuring each factor is provided, along with justification of the importance in recording and stating each factor.

| **Factor** | **Potential measures** | **Justification** |
| --- | --- | --- |
| **Carcass selection** | | |
| Size | Weight (kg). | Carcass size influences scavenger assemblages, species richness, biomass loss and detection time (eg Matuszewski *et al.* 2014; Moleón *et al.* 2015). |
| Type | Species, dietary habits (e.g. carnivore/herbivore). | Carnivore carcasses may repel herbivores (eg Munoz-Lozano *et al.* 2019). |
| Condition | Fresh, frozen. | Freezing a carcass can affect the decomposition process, internal microbial communities, and insect succession (eg Micozzi 1986). |
| State | Whole, open, in parts. | Whole, unopened carcasses are less likely to be utilised by scavengers than open carcasses or carcasses in parts (eg Read and Wilson 2004; Selva *et al.* 2005). Insects also target open wounds for oviposition, leading to increased maggot activity (eg Munro *et al.* 2019) |
| Source | Road kill, hunter shot, store bought, predator kill. | See “State” and “Condition” factors above. Carcasses often come in parts or are opened in some way depending on the type of death. |
| Time of day placed | Time. | Depositing carcasses in the morning versus the evening can attract different rates of scavenging by different species e.g. nocturnal versus diurnal species (eg Carrasco-Garcia *et al.* 2018), and some flies may not oviposit during the night (eg Williams *et al.* 2017) |
| Carcass age | Days since death. | Carcass age will determine the quality of the tissues (eg moisture content) (eg Barton *et al.* 2019) and what stage of succession the associated insect and microbe community has reached (eg Pechal *et al.* 2019). |
| **Site/season selection** | | |
| Biome/ landscape | e.g. Desert, alpine, temperate forest. | Biome influences the structure of scavenger communities in a system (eg Pardo-Barquin et al. 2019). |
| Habitat (micro/macro scale) | Open, closed. | Scavenger presence and carrion detection may vary between open and closed environments with avian species more typically dominant in more open environments (eg Carrasco-Garcia *et al.* 2018; Pardo-Barquin *et al.* 2019) |
| Location/ position details | Buried, ground, arboreal etc. | Carcasses buried will prevent access by insect scavengers and slow decomposition rates. Carcasses placed in trees may be exposed to different scavenger assemblages. |
| Human disturbance | Human impact index. | Scavenger species richness is lowest in areas of high human impact (eg Sebastián‐González *et al.* 2019). |
| Season | Exact date, exact season (i.e. spring, summer, autumn, winter), often grouped into “warm” and “cool” seasons. | Season affects scavenger composition, and carrion persistence (eg Benbow *et al.* 2013; Turner *et al.* 2017). |
| **Abiotic measures** | | |
| Temperature | Daily temperature record. | Temperature and humidity affects biochemical processes, and microbial and insect activity (eg Barton and Bump 2019). |
| Humidity/ rainfall | Daily humidity record/ daily rainfall record (mm). | Changes in moisture may speed or slow carcass decomposition rates (eg Matuszewski *et al.* 2010). |
| Snow cover | Depth (cm). | May increase or change scavenging by certain vertebrate species (eg Selva *et al.* 2005). |

**References**

Barton, P.S., Bump, J.K., 2019. Carrion decomposition, in: Olea, P.P., Mateo-Tomás, P., Sánchez Zapata, J.A. (Eds.), Carrion Ecology and Management. Springer International Publishing, Switzerland AG, pp. 101–124.

Barton, P.S., Strong, C., Evans, M.J., Higgins, A., Quaggiotto, M.-M., 2019. Nutrient and moisture transfer to insect consumers and soil during vertebrate decomposition. Food Webs 18, e00110. https://doi.org/10.1016/j.fooweb.2018.e00110

Benbow, M.E., Lewis, A.J., Tomberlin, J.K., Pechal, J.L., 2013. Seasonal Necrophagous Insect Community Assembly During Vertebrate Carrion Decomposition. J. Med. Entomol. 50, 440–450. https://doi.org/10.1603/ME12194

Carrasco-Garcia, R., Barroso, P., Perez-Olivares, J., Montoro, V., Vicente, J., 2018. Consumption of big game remains by scavengers: A potential risk as regards disease transmission in central Spain. Front. Vet. Sci. 5, 4. https://doi.org/10.3389/fvets.2018.00004

Matuszewski, S., Bajerlein, D., Konwerski, S., Szpila, K., 2010. Insect succession and carrion decomposition in selected forests of Central Europe. Part 1: Pattern and rate of decomposition. Forensic Sci. Int. 194, 85–93. https://doi.org/10.1016/j.forsciint.2009.10.016

Matuszewski, S., Konwerski, S., Frątczak, K., Szafałowicz, M., 2014. Effect of body mass and clothing on decomposition of pig carcasses. Int. J. Legal Med. 128, 1039–1048. https://doi.org/10.1007/s00414-014-0965-5

Micozzi, M.S., 1986. Experimental study of postmortem change under field conditions: Effects of freezing, thawing, and mechanical injury. J. Forensic Sci. 31, 11103J. https://doi.org/10.1520/JFS11103J

Moleón, M., Sánchez-Zapata, J.A., Sebastián-González, E., Owen-Smith, N., 2015. Carcass size shapes the structure and functioning of an African scavenging assemblage. Oikos 124, 1391–1403. https://doi.org/10.1111/oik.02222

Munoz-Lozano, C., Martin-Vega, D., Martinez-Carrasco, C., Sanchez-Zapata, J.A., Morales-Reyes, Z., Gonzalvez, M., Moleon, M., 2019. Avoidance of carnivore carcasses by vertebrate scavengers enables colonization by a diverse community of carrion insects. PLOS ONE 14, e0221890. https://doi.org/10.1371/journal.pone.0221890

Munro, H.L., Mondor, E.B., Lampert, E.C., 2019. Does sharp force trauma alter blow fly attraction to, colonization of, and decomposition of vertebrate remains? Entomol. Exp. Appl. 167, 490–499. https://doi.org/10.1111/eea.12767

Pardo-Barquin, E., Mateo-Tomas, P., Olea, P.P., 2019. Habitat characteristics from local to landscape scales combine to shape vertebrate scavenging communities. BASIC Appl. Ecol. 34, 126–139. https://doi.org/10.1016/j.baae.2018.08.005

Pechal, J.L., Crippen, T.L., Cammack, J.A., Tomberlin, J.K., Benbow, M.E., 2019. Microbial communities of salmon resource subsidies and associated necrophagous consumers during decomposition: Potential of cross-ecosystem microbial dispersal. Food Webs 19, e00114. https://doi.org/10.1016/j.fooweb.2019.e00114

Read, J.L., Wilson, D., 2004. Scavengers and detritivores of kangaroo harvest offcuts in arid Australia. Wildl. Res. 31, 51–56. https://doi.org/10.1071/WR02051

Sebastián‐González, E., Barbosa, J.M., Pérez‐García, J.M., Morales‐Reyes, Z., Botella, F., Olea, P.P., Mateo‐Tomás, P., Moleón, M., Hiraldo, F., Arrondo, E., Donázar, J.A., Cortés‐Avizanda, A., Selva, N., Lambertucci, S.A., Bhattacharjee, A., Brewer, A., Anadón, J.D., Abernethy, E., Rhodes, O.E., Turner, K., Beasley, J.C., DeVault, T.L., Ordiz, A., Wikenros, C., Zimmermann, B., Wabakken, P., Wilmers, C.C., Smith, J.A., Kendall, C.J., Ogada, D., Buechley, E.R., Frehner, E., Allen, M.L., Wittmer, H.U., Butler, J.R.A., du Toit, J.T., Read, J., Wilson, D., Jerina, K., Krofel, M., Kostecke, R., Inger, R., Samson, A., Naves‐Alegre, L., Sánchez‐Zapata, J.A., 2019. Scavenging in the Anthropocene: human impact drives vertebrate scavenger species richness at a global scale. Glob. Change Biol. gcb.14708. https://doi.org/10.1111/gcb.14708

Selva, N., Jędrzejewska, B., Jędrzejewski, W., Wajrak, A., 2005. Factors affecting carcass use by a guild of scavengers in European temperate woodland. Can. J. Zool. 83, 1590–1601.

Turner, K.L., Abernethy, E.F., Conner, L.M., Rhodes, O.E., Beasley, J.C., 2017. Abiotic and biotic factors modulate carrion fate and vertebrate scavenging communities. Ecology 98, 2413–2424. https://doi.org/10.1002/ecy.1930

Williams, K.A., Wallman, J.F., Lessard, B.D., Kavazos, C.R.J., Mazungula, D.N., Villet, M.H., 2017. Nocturnal oviposition behavior of blowflies (Diptera: Calliphoridae) in the southern hemisphere (South Africa and Australia) and its forensic implications. Forensic Sci. Med. Pathol. 13, 123–134. https://doi.org/10.1007/s12024-017-9861-x
